# Supplementary material for: Identification and Characterization of Bacteria-Derived Antibiotics for the Biological Control of Pea Aphanomyces Root Rot
Source: Microorganisms. 2022 Aug 8;10(8):1596. doi: 10.3390/microorganisms10081596 (PMC9416638; doi:10.3390/microorganisms10081596)
Supplement: Supplementary file 1 [file microorganisms-10-01596-s001.zip › microorganisms-1841804-supplementary.pdf]

**Table S1.** Bacterial strains isolated in this study and their identities to the most closely related strains found in the NCBI database.

| **Bacterial Strains*** | **Closely related strain from NCBI database*** | **Identity** |
|---|---|---|
| 39_109 *Pseudomonas* sp. (ON885283) | *Pseudomonas salomonii* (LC486826) | 99.85% |
| 40_101 *Pseudomonas* sp. (ON885284) | *Pseudomonas synxantha* (MN559446) | 99.93% |
| 2_4 *Pseudomonas* sp. (ON885280) | *Pseudomonas cedrina* (MK737138) | 99.78% |
| 39_99 *Pseudomonas* sp. (ON885281) | *Pseudomonas frederiksbergensis* (MK849865) | 99.71% |
| 1_1 *Bacillus* sp. (ON885277) | *Bacillus atrophaeus* (NR_112723) | 99.86% |
| 16_134 *Paenibacillus* sp. (ON885278) | *Paenibacillus polymyxa* (FJ940900) | 99.78% |
| 23_252 *Paenibacillus* sp. (ON885282) | *Paenibacillus polymyxa* (MT367718) | 99.71% |
| 42_176 *Paenibacillus* sp. (ON885279) | *Paenibacillus polymyxa* (KU860093) | 100% |
| 3_143 *Pseudarthrobacter* sp. (ON945547) | *Pseudarthrobacter equi* (NR_117032) | 99.56% |
| 45_162 *Chryseobacterium* sp. (ON885285) | *Chryseobacterium vrystaatense* (AJ871398) | 99.21% |

* Accession number is shown in the parentheses.

## Supplementary Figure S1

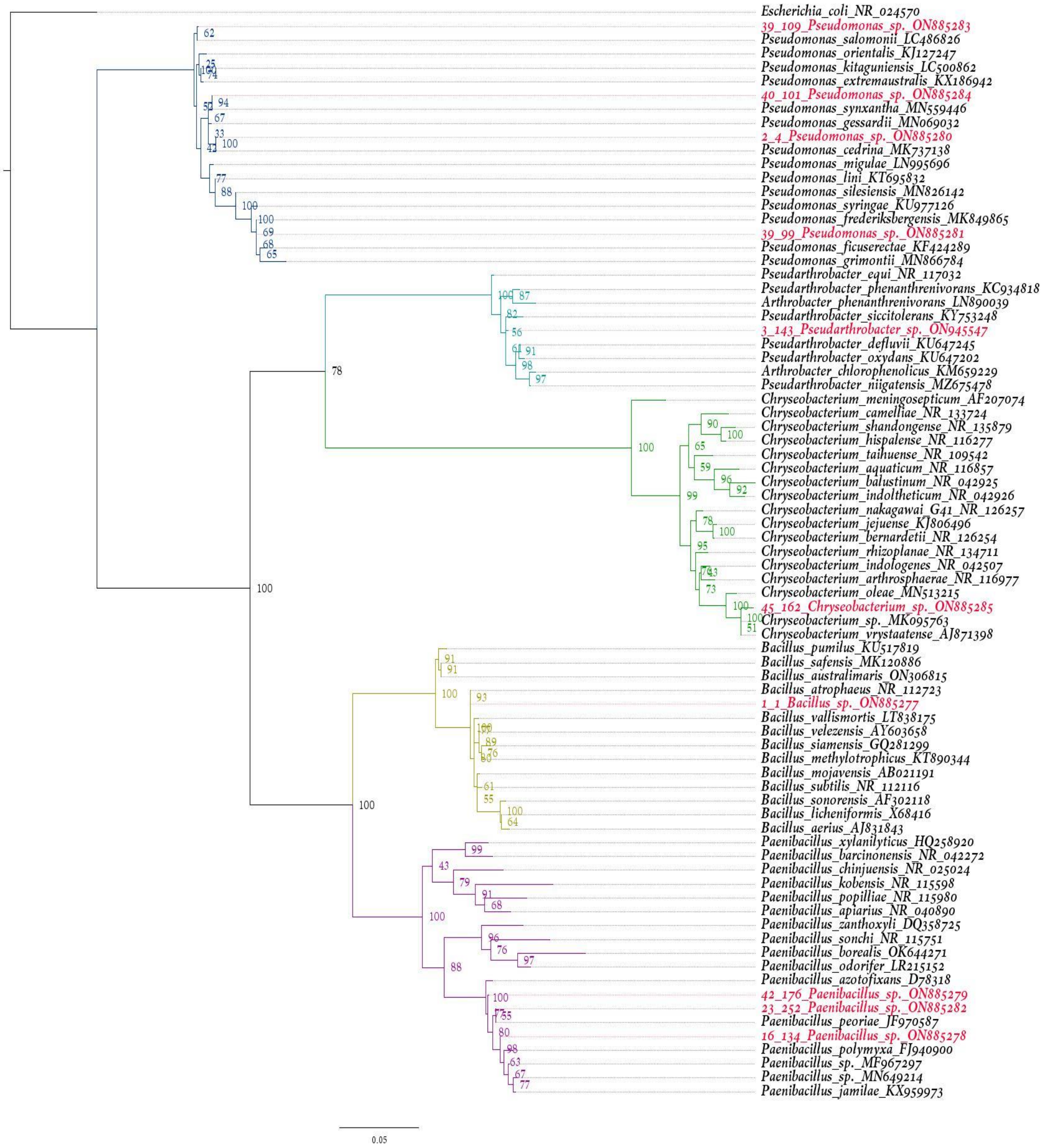


**Figure S1.** Phylogenetic analysis of the bacterial antagonists isolated in this study. The taxonomy of the bacterial antagonists was identified by 16S rRNA analysis. The phylogenetic tree was constructed using IQ-tree and visualized using Figtree v 1.4.4. Five different bacterial genera forming distinct clades are represented by different colors at the branches. The ten bacterial isolates highlighted with maroon color were isolated in this study. The GenBank accession number of each isolate was shown. The values at the node of the tree represent bootstrap support. *Escherichia coli* (NR_024570) was used as an outgroup bacterial strain. The scale at the bottom represents nucleotide substitution per site.
